# Chitosan and its derivatives regulate lactic acid synthesis during milk fermentation

**DOI:** 10.3389/fnut.2024.1441355

**Published:** 2024-09-16

**Authors:** Vladimir Kurchenko, Tatsiana Halavach, Alexey Yantsevich, Mariya Shramko, Lyudmila Alieva, Ivan Evdokimov, Alexey Lodygin, Vladimir Tikhonov, Andrey Nagdalian, Faten M. Ali Zainy, Ammar AL-Farga, Nora Abdullah ALFaris, Mohammad Ali Shariati

**Affiliations:** ^1^Department of Biology, Belarusian State University, Minsk, Belarus; ^2^Laboratory of Food and Industrial Biotechnology, Faculty of Food Engineering and Biotechnology, North Caucasus Federal University, Stavropol, Russia; ^3^Institute of Bioorganic Chemistry of the National Academy of Sciences of Belarus, Minsk, Belarus; ^4^Laboratory of Heterochain Polymers, A.N. Nesmeyanov Institute of Organoelement Compounds of Russian Academy of Sciences, Moscow, Russia; ^5^Department of Biochemistry, College of Sciences, University of Jeddah, Jeddah, Saudi Arabia; ^6^Department of Physical Sports Sciences, College of Sports Sciences and Physical Activity, Education, Princess Nourah Bint Abdulrahman University, Riyadh, Saudi Arabia; ^7^Scientific Department, Semey Branch of the Kazakh Research Institute of Processing and Food Industry, Almaty, Kazakhstan

**Keywords:** oligochitosan, lactic acid, lactobacilli, fatty acids, propanol, benzaldehyde, secondary metabolites, glucosamine monomers

## Abstract

**Introduction:**

The influence of chitosan's physicochemical characteristics on the functionality of lactic acid bacteria and the production of lactic acid remains very obscure and contradictory to date. While some studies have shown a stimulatory effect of oligochitosans on the growth of Lactobacillus spp, other studies declare a bactericidal effect of chitosan. The lack and contradiction of knowledge prompted us to study the effect of chitosan on the growth and productivity of *L. bulgaricus* in the presence of chitosan and its derivatives.

**Methods:**

We used high molecular weight chitosan (350 kDa) and oligochitosans (25.4 and 45.3 kDa). The experiment was carried out with commercial strain of *L. bulgaricus* and the low fat skim cow milk powder reconstituted with sterile distilled water. After fermentation, dynamic viscosity, titratable acidity, pH, content of lactic acid, colony forming units, chitosan and oligochitosans radii were measured in the samples. Fermented dairy products were also examined using sodium dodecyl sulfate electrophoretic analysis, gas chromatography-mass spectrometry and light microscopy.

**Results and discussion:**

The results of the study showed that when *L. bulgaricus* was cultured in the presence of 25.4 kDa oligochitosans at concentrations of 0.0025%, 0.005%, 0.0075% and 0.01%, the average rate of LA synthesis over 24 hours was 11.0 × 10^−3^ mol/L/h, 8.7 × 10^−3^ mol/L/h, 6.8 × 10^−3^ mol/L/h, 5.8 × 10^−3^ mol/L/h, respectively. The 45.3 kDa oligochitosans had a similar effect, while the average rate of lactic acid synthesis in the control sample was only 3.5 × 10^−3^ mol/L/h. Notably, 350 kDa chitosan did not affect the rate of lactic acid synthesis compared with the control sample. Interestingly, interaction of chitosan with *L. bulgaricus* led to a slowdown in the synthesis of propanol, an increase in the content of unsaturated and saturated fatty acids, and a change in the composition and content of other secondary metabolites. The quantity of *L. bulgaricus* in a sample with 0.01% chitosan exceeded their content in the control sample by more than 1,700 times. At the same chitosan concentration, the fermentation process was slowed down, increasing the shelf life of the fermented milk product from 5 to 17 days while maintaining a high content of *L. bulgaricus* (6.34 × 10^6^ CFU/g).

## 1 Introduction

A significant amount of scientific research has been dedicated to improving the quality and functional value of food products over the last few decades, as functional products can enhance people's quality of life and health compared to conventional foods (1, 2). One of the most common groups of functional food products includes fermented dairy products, which are recognized as the most popular and extensively produced and consumed dairy products worldwide (3, 4). In the production of fermented dairy products, the production of lactic acid by bacteria of the genera *Lactobacillus* and *Bifidobacterium genera* is commonly employed (5). Among lactic acid bacteria, *L. bulgaricus* represents the most widespread in Central, Eastern, and South-Eastern Europe, commonly used in the preparation of fermented dairy products (6). During the growth of *L. bulgaricus* in milk and dairy products, the bacteria produce beta-β-galactosidase (E.C.3.2.1.23), which catalyzes the fermentative cleavage of milk lactose (β-D-galactopyranosyl-(1 → 4)-D-glucose) into glucose and galactose, followed by the homofermentation of these carbohydrates into lactic acid as the sole or main end product (7, 8).

The cultivation of lactic acid bacteria usually leads to the production of functional additives such as macro- and micronutrients and dietary fiber (9, 10). Among them, natural poly- and oligosaccharides are of particular interest and value, as they can be used not only as thickeners and gelling agents but also as prebiotics that stimulate the growth of beneficial microorganisms and prolongs their activity in the digestive system (11, 12). Considering this, it is important to note that recent works are increasingly studying Chitosan as polysaccharides with specific properties (13–15).

Chitosan is a partially or fully deacetylated chitin—poly-(1 → 4)-β-D-N-acetylglucosamine. It is produced industrially by the deacetylation of chitin from crab, shrimp, or insect shells and is widely used in pharmaceuticals, food, and cosmetic compositions due to its wide range of antimicrobial activities against bacteria, molds, and yeasts (16–19). As an artificial biopolymer, chitosan is characterized by its molecular weight and degree of deacetylation (%) (20, 21). Based on molecular weight, chitosan can be divided into three categories: low molecular weight (molecular weight <150 kDa), medium molecular weight (molecular weight <700 kDa), and high molecular weight (molecular weight > 700 kDa) (22).

High molecular weight chitosan possesses longer molecular chains with the availability of more hydroxyl groups (23). There is also a higher possibility that there are more amino groups, although the number of amino groups is determined by the degree of deacetylation (24). High molecular weight, high degree of polymerization, and, as a consequence, a high number of inter- and intra-molecular hydrogen bonds inside the polymer chains determine the relatively low solubility of High molecular weight chitosan and limit its application in some products due to high viscosity (25). However, specific properties of high molecular weight chitosan found wide application in development of active films for food packaging application (26). Medium molecular weight chitosan is soluble in weak acid solutions. This limits their usage compared to the antimicrobial capacity of acid- and water-soluble chitosan with different degrees of deacetylation and viscosities (22). Low molecular weight chitosan is a linear amino polysaccharide with high nitrogen content. It is a weak base with deprotonated amino groups as nucleophiles that is able to form hydrogen bonds between molecules and has highly reactive groups for crosslinking and chemical activation (27).

Low molecular weight chitosan forms salts with organic and inorganic acids, has chelating and complex properties, and exhibits ionic conductivity as polyelectrolytes (pH <7) (28). In contrast to high and medium molecular weight chitosan, low molecular weight chitosan demonstrates considerable solubility in various media. However, it is characterized by an unpredictably wide distribution of molecular weight and degree of deacetylation, complicating the standardization of parameters for industrial applications (29–31). Therefore, to address this challenge in dispersing food systems such as milk, a promising avenue lies in the conversion of chitosan into oligomers with more consistent molecular sizes and improved solubility (32). These oligomers, termed oligochitosans, result from the profound depolymerization of chitosan. It is noteworthy that significant disparities exist in the physicochemical parameters and biological activity between chitosan and oligochitosans, accentuating the scientific interest in their comparative study within the realm of food technology applications (33–35).

Amidst its bioavailability, safety, and antimicrobial attributes, chitosan exhibits immunostimulatory and anti-angiogenic properties, mitigating the risk of neurodegenerative conditions while facilitating the regeneration of articular cartilage in osteoarthritis, and enhancing the bioavailability of glucosamine (36–38). In an acid media, chitosan has a high positive charge density due to the protonation of free amino groups and can interact with negatively charged cell walls (31, 39). Therefore, chitosan can potentially interact with the plasma membrane of lactic acid bacteria causing a stress and perturbation of the membrane walls and the death of the cells (40, 41). As a response, LAB can generate a number of adaptive reactions (42, 43) which may insert desirable or undesirable changes in the functionality of lactic acid bacteria and fermented dairy products.

Unfortunately, the influence of chitosan's physicochemical characteristics on the functionality of lactic acid bacteria and the production of lactic acid remains very obscure and contradictory to date. While some studies have shown a stimulatory effect of oligochitosans on the growth of *Lactobacillus* spp., which used added oligochitosans as nutrients (44, 45), others have described a bactericidal effect of chitosan (40, 42, 46). The lack and contradiction of knowledge prompted us to study the effect of chitosan on the growth and productivity of *L. bulgaricus* in the presence of chitosan and its derivatives. The aim of this work was therefore to study the effect of different concentrations of oligochitosans and chitosan on the production of lactic acid and secondary metabolites by *L. bulgaricus* during fermentation and long-term storage of skimmed cow's milk.

## 2 Materials and methods

### 2.1 Materials

Commercial strain of *L. bulgaricus* and the low fat skim cow milk powder (solubility−93%, moisture content−5%; fat content−1.5%; proteins−32%, and lactose−50 ± 3%) were purchased from official store of “Stavropolsky Dairy Plant” (Stavropol, Russia). The milk powder was reconstituted with sterile distilled water (50 ml) at temperature 30–35°C during 24 h at sterile conditions to the total solids concentration not <9.0%. The reconstituted skim milk solution was characterized by the following parameters: proteins−3.2%, lactose−5%, total fat−0.15%, and mineralization−0.7%, pH 5.9.

High molecular weight chitosan with 350 kDa molecular weight and 5.0% DD manufactured by Bioprogress Ltd. (Moscow, Russia). Two types of oligochitosans hydrochlorides with 25.4 kDa molecular weight, 1.0% degree of deacetylation and 45.3 kDa molecular weight, 1.5% degree of deacetylation were obtained from the initial chitosan according to the protocol of Berezin et al. (47). The obtained oligochitosans were analyzed in accordance with the requirements of the EU Pharmacopeia 9.0 for chitosan hydrochloride (48). Oligochitosans with a solubility in demineralized water Milli-Q > 99.95% formed a colorless and untroubled 1% solution in accordance with the tests on transparency and degree of opalescence, and degree of liquids coloring. The initial 1% chitosan solution was prepared by dissolving the sample in 1% lactic acid. The pH of the initial solution of the sample was adjusted to pH 5.0 by adding drops of 1M sodium hydroxide solution.

### 2.2 Milk sample preparation and fermentation

Reconstituted skim milk (100 mL) was mixed with a fixed amount (0.0025, 0.005, 0.075, and 0.01 g) of chitosan stock solution, and the mixture was pasteurized at 85°C for 5 min. After cooling to 43–45°C, 3 g of the commercial freeze-dried starter culture of *L. bulgaricus* was added to achieve a viable count of 10^5^ CFU/mL in the sample. The sample was thoroughly mixed and incubated at 43–45°C for 17 days until the maximum titratable acidity was reached. Subsequently, the sample was stored at 4°C before being analyzed.

### 2.3 Fermented milk product analysis

#### 2.3.1 Dynamic viscosity

After the storage, the dynamic viscosity of fermented dairy product was measured at 20°C using a Brookfield digital rotational viscometer DV-II+PRO (Brookfield Engineering Laboratories, Middleboro, MA, USA).

#### 2.3.2 Titratable acidity and pH

During the fermentation, the milk sample (10 mL) was centrifuged at 6000 rpm (30 min) using MicroCL 17R centrifuge (Thermo FS, Waltham, MA, USA), and pH value of the supernatant was measured with analizator Expert-001 (Econix-Expert, Moscow, Russia). The titratable acidity of fermented dairy product was determined by titration of the supernatant with 0.1 N NaOH using phenolphthalein as indicator (49). Titratable acidity was expressed in percentages of lactic acid content (T, %) or as the volume of 0.1 M of sodium hydroxide consumed for the neutralization of 100 ml of fermented milk product (V_t_, mL).

#### 2.3.3 Content of lactic acid

Molar concentrations of lactic acid (mol/L) in the fermented milk supernatant were determined following the method described in the previous work (50). The molar concentrations of lactic acid (mol/L) were calculated following the equation:


(1) $$\begin{array}{l} LA\left(mol/L\right)=\left[V_{\text{t}}\;0.1\right]/100; \end{array}$$

where V_t_–titratable acidity of the fermented milk supernatant.

#### 2.3.4 Sodium dodecyl sulfate electrophoretic analysis

The analysis of the protein composition of fermented milk product was carried out after 17 days of storage using the method of sodium dodecyl sulfate (SDS) electrophoretic separation in polyacrylamide gel (20%) under denaturing conditions (DSN electrophoresis) in accordance with the generally accepted protocol (51).

#### 2.3.5 Colony forming units counting

Colony count technique for CFU determination was used in accordance with ISO 15214 (1998-2021) after 10–1000-fold dilution of the fermented milk supernatant solution.

#### 2.3.6 Microscopy

Sample supernatant was centrifuged at 15,000 rpm for 10 min using MicroCL 17R centrifuge (Thermo FS, Waltham, MA, USA) and filtrated using a 0.2 μm PVDF membrane. The morphology of lactic acid bacteria was observed after the membrane filtration. Samples was treated with methylene blue, destained by water washing and dried on air. Photographs were made and recorded using a BIOLAM light microscope (Scopica, Ekaerinburg, Russia).

#### 2.3.7 Measuring the chitosan and oligochitosans radii

The dynamic light scattering method was used to measure the chitosan and oligochitosans molecules radii. Experiments on dynamic light scattering were carried out in a disposable micro-cuvette with a volume of 4 μl, using a detector located at an angle of 90° in the DynaPro Nanostar device (Wyatt Technology, Santa Barbara, CA, USA). The samples were filtered through a 0.2 μm nylon filter. The data was analyzed using the “Regulation fit” (multimodal) analysis method in the Dynamics software (Wyatt Technology, Santa-Barbara, CA, USA).

#### 2.3.8 Analysis of secondary metabolites: gas chromatography-mass spectrometry

After 17 days of storage, a 10 mL sample of the fermented dairy product was centrifuged at 6,000 rpm for 30 min using a MicroCL 17R centrifuge (Thermo FS, Waltham, MA, USA), followed by lyophilization. The residual solid product (1 g) was then extracted twice with 70% ethanol (1:10 wt/v). The resulting extracts were combined and filtered through 0.25 μm PVDF membranes. Secondary metabolites were separated and analyzed using an Agilent 5975B gas chromatograph equipped with a mass selective detector (Agilent Technologies, Santa Clara, CA, USA). The separation of secondary metabolites was performed using a capillary column DB-5MS (5% phenyl methyl siloxane, J&W 122-5062). Identification of components in the mass spectra was conducted using a library of mass spectra NIST0.5A. Comparative semi-quantitative analysis of secondary metabolites was performed based on peak areas without using correction factors. The semi-quantitative content of secondary metabolites was calculated from the peak area without considering the peak of lactic acid and without using correction factors.

### 2.4 Statistical analysis

In order to compare the means of factor's levels, a one-, two-, or three-way analysis of variance (ANOVA) with subsequent Dunnett's test (comparing several treatments with a control), Student's *t*-test (matching the means of two groups) or Tukey's Honest Significant Difference (HSD) test (performing multiple pairwise comparisons) were applied. R functions aov, DunnettTest, *t*-test, TukeyHSD and DescTools packages were involved in the statistical analysis. Statistical differences between groups were set as significant at *p* < 0.05 level with correction for multiple pairwise comparisons. Correlation analysis was performed using Pearson's Criterion. Plots were created in Microsoft Office Excel (MS Corporation, Shadeland, IN, USA). The data in tables and graphs are shown as the mean ± the half-width of 95% confidence interval (*n* = 3).

## 3 Results and discussion

### 3.1 Cultivation of *L. bulgaricus* in the presence of chitosan and oligochitosans

**Figure 1:**
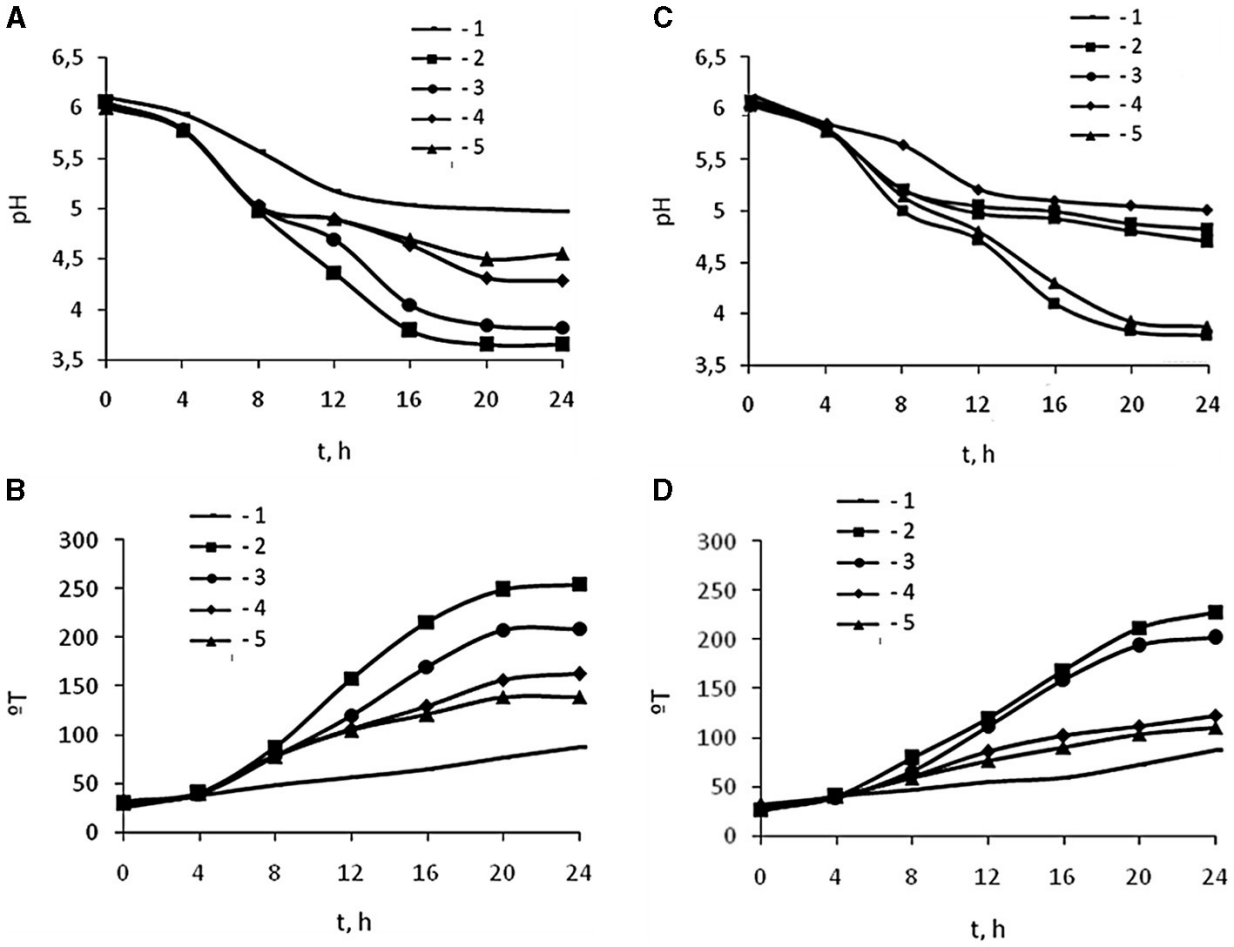
Effect 25.4 kDa **(A, B)** and 45.3 kDa **(C, D)** OCHs on active acidity pH **(A, C)** and titratable acidity °T **(B, D)** during 24 h cultivation (t, h) of *L. bulgaricus*: 1 – control sample, 2 – 0.0025 % OCHs, 3 – 0.0050 % OCHs, 4 – 0.0075 % OCHs, 5 – 0.01 % OCHs.

The effect of oligochitosans of 25.4 kDa molecular weight, 1% degree of deacetylation, and 45.3 kDa molecular weight, 1.5% degree of deacetylation on the lactic acid fermentation process was studied in comparison with CH of 350 kDa molecular weight, 5.0% degree of deacetylation at *L. bulgaricus* cultivation. During cultivation, pH and titratable acidity of the culture liquid were measured in experimental and control samples every 4 h for 24 h. Based on the results of titratable acidity, the molar concentration and the rate of synthesis of lactic acid were calculated. Figure 1 shows changes in the pH and titratable acidity of a culture liquid containing 0.0025, 0.005, 0.0075, and 0.01% oligochitosans of 25.4 and 45.3 kDa. According to the data obtained, pH depends on the concentration of oligochitosans (Figures 1A, C). Notably, the maximum decrease in pH was observed at oligochitosans concentration of 0.0025% (Table 1). At the end of 24-h cultivation, pH of the experimental samples was lower than in control sample by 73% in 25.4 kDa oligochitosans, by 75% in 45.3 kDa oligochitosans, and by 1% in 350 kDa chitosan. At higher concentrations, pH less intense decreased in samples with oligochitosans, but particularly was not changed in samples with chitosan. Inversely dependent, the titratable acidity of the culture liquid decreased with the increase of oligochitosans concentration, but was higher than the titratable acidity of control samples (Figures 1B, D).

**Table 1:** Effect of OCHs and CH on LA fermentation parameters after 24 h of *L. bulgaricus* cultivation.

**Index**	**Concentration of OCHs and CH, %**				
	**0 (control)**	**0.0025**	**0.005**	**0.0075**	**0.01**
**25.4 kDa, 1% DD OCH (glucosamine monomers 140/mol OCH)**					
Molar concentration of OCH, mol/L	-	0.99 × 10^−6^	1.98 × 10^−6^	2.98 × 10^−6^	3.97 × 10^−6^
Glucosamine monomers content, mol/L	-	1.39 × 10^−4^	2.78 × 10^−4^	4.17 × 10^−4^	5.56 × 10^−4^
pH	5.00 ± 0.20^A^	3.65 ± 0.10^B^	3.82 ± 0.10 ^B^	4.29 ± 0.10 ^C^	4.56 ± 0.20 ^C^
LA content, mol/L	0.085 ± 0.009^A^	0.255 ± 0.02^B^	0.209 ± 0.019^C^	0.163 ± 0.015^D^	0.139 ±0.011^E^
The average rate of LA synthesis, mol/L/h	3.5 × 10^−3*A, I*^	11.0 × 10^−3*B, I*^	8.7 × 10^−3*C, I*^	6.8 × 10^−3*D, I*^	5.8 × 10^−3*E, I*^
**45.3 kDa, 1.5% DD OCH (glucosamine monomers 248/mol OCH)**					
Molar concentration of OCH, mol/L	-	0.56 × 10^−6^	1.12 × 10^−6^	1.68 × 10^−6^	2.24 × 10^−6^
Glucosamine monomers content, mol/L	-	1.39 × 10^−4^	2.78 × 10^−4^	4.17 × 10^−4^	5.56 × 10^−4^
pH	5.00 ± 0.20^A^	3.78 ± 0.10^B^	3.87 ± 0.10^B^	4.70 ± 0.20^C^	4.82 ± 0.10^C^
LA content, mol/L	0.087 ± 0.009^A^	0.228 ± 0.025^B^	0.202 ± 0.018^B^	0.123 ± 0.011^C^	0.111 ± 0.008^C^
The average rate of LA synthesis, mol/L/h	3.6 × 10^−3*A, I*^	9.5 × 10^−3*B, I*^	8.4 × 10^−3*B, I*^	5.1 × 10^−3*C, II*^	4.6 × 10^−3*C, II*^
**350 kDa, 5% DD CH (glucosamine monomers 1850/mol CH)**					
Molar concentration of CH, mol/L	-	0.75 × 10^−7^	1.50 × 10^−7^	2.25 × 10^−7^	3.00 × 10^−7^
Glucosamine monomers content, mol/L	-	1.39 × 10^−4^	2.78 × 10^−4^	4.17 × 10^−4^	5.56 × 10^−4^
pH	5.00 ± 0.20^A^	4.95 ± 0.20^A^	4.99 ± 0.10^A^	4.98 ± 0.10^A^	5.01 ± 0.20^A^
LA content, mol/L	0.090 ± 0.010^A^	0.089 ± 0.009^A^	0.082 ± 0.009^B^	0.083 ± 0.009^B^	0.075 ± 0.007^C^
The average rate of LA synthesis, mol/L/h	3.8 × 10^−3*A, I*^	3.7 × 10^−3*A, II*^	3.4 × 10^−3*B, II*^	3.4 × 10^−3*B, III*^	3.1 × 10^−3*C, III*^

The values represent the mean ± SD (n = 3). Means without a common letter within the same column (I–III) and row (A–E) indicate significant difference at p <0.05.

According to Table 1, at the same percentage concentration of oligochitosans and chitosan, their molar concentration in fermented dairy products differs significantly. However, the total content of glucosamine monomers included in their composition was the same. In the acidic environment, the oligochitosans amino groups are in a protonated state (52). Being positive charge, they can interact with negatively charged cell walls of lactic acid bacteria (53, 54). With an increase in the concentration of oligosaccharides in the experimental samples, the content of glucosamine monomers also increased. Consequently, the number of positively charged amino groups capable of interacting with lactic acid bacteria increased.

It was revealed that at 0.0025% concentration and 1.39 × 10^−4^ mol/L glucosoamine monomers, oligochitosans increase the synthesis of lactic acid by 3 times (25.4 kDa) and 2.7 times (45.3 kDa) relative to control sample. It should be noted that samples with chitosan, like samples with oligochitosans, contained 1.39 × 10^−4^ mol/L glucosoamine monomers at a concentration of 0.0025%. However, it was surprisingly found that at 0.0025% chitosan and 1.39 × 10^−4^ mol/L glucosoamine monomers, chitosan slowed down the synthesis of lactic acid by 0.98 times relative to the control. Higher concentrations of chitosan more intense affected lactic acid synthesis and consequently, fermented dairy product with chitosan had less lactic acid concentration than control sample.

**Figure 2:**
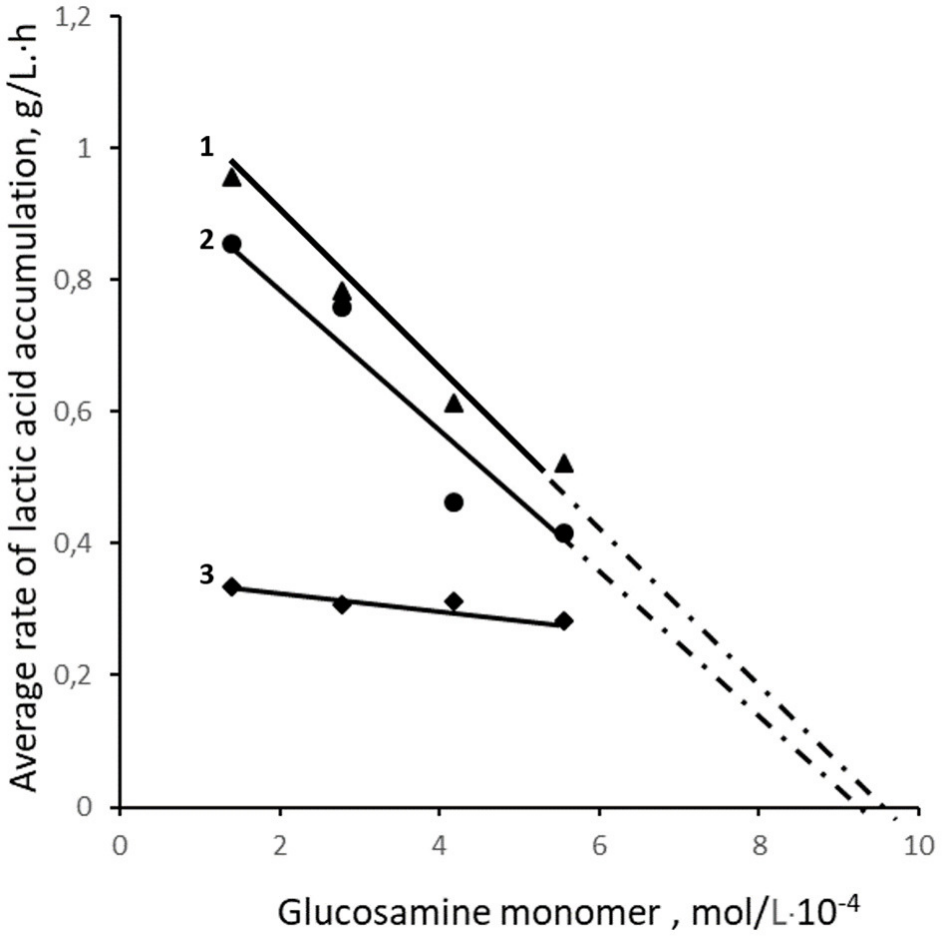
Effect of the content of glucosamine monomers in samples with 25.4 kDa OCHs (1), 45.3 kDa OCHs (2), and 350 kDa CH (3) on the average rate of LA synthesis.

On the other hand, at the highest concentration of oligochitosans (0.01 %), the content of glucosamine monomers reached 5.56 × 10^−4^ mol/L and lactic acid synthesis slowed down. However, the resulting lactic acid content in both oligochitosans groups was still higher than in the control sample. The rate of lactic acid synthesis is critically important in milk fermentation (55). Thus, dependence of the average rate of lactic acid synthesis on concentration of glucosamine monomers of oligochitosans and chitosan at 24 h-cultivation was analyzed and presented in Figure 2.

**Figure 3:**
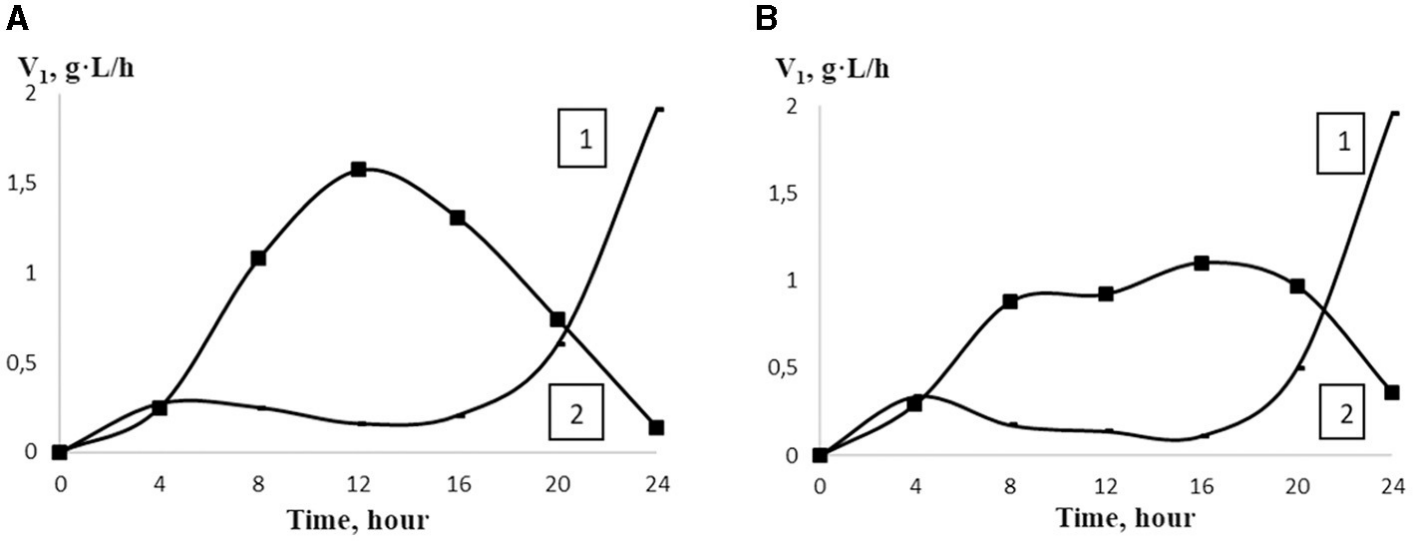
The rate of LA accumulation (V_1_) in the control sample (1) and in the presence of 25.4 kDa OCHs **(A)** and 45.3 kDa OCHs **(B)** at concentration of 0.0025% (2) during 24 h cultivation of *L. bulgaricus* (t).

The analysis of the interaction of lactic acid bacteria with the positively charged amino groups of oligochitosans glucosamine monomers shows that with an increase in their concentration, the rate of lactic acid synthesis slows down (Figure 2). Theoretically, at a concentration of oligochitosans glucosamine monomers close to 10^−3^ mol/L, a process of deep inhibition of lactose metabolism and lactic acid synthesis is possible (56, 57). Similarly, an increase in the content of glucosamine monomers of 350 kDa chitosan leads to a reduction in lactic acid, as shown in Figure 2. According to Figure 1, pH and titratable acidity stabilize on the 20th h of cultivation in samples with 0.0025% oligochitosans. This indicates a slowdown in the synthesis of lactic acid by lactic acid bacteria. Therefore, it is critically important to understand the dynamics of lactic acid accumulation in the presence of oligochitosans. For this purpose, the rate of lactic acid synthesis was analyzed every 4 h of cultivation. The results obtained are presented in Figure 3.

Notably, the maximum rate of lactic acid synthesis was observed at 0.0025% 25.4 kDa oligochitosan on the 12th h of *L. bulgaricus* cultivation, which is 6.44 times higher than in the control sample. Similarly, on the 12th h of *L. bulgaricus* cultivation, the rate of lactic acid synthesis was the most intense, being 5.44 times higher than in the control sample. It should be pointed out that with an increase in oligochitosans concentration, the rate of lactic acid synthesis decreases and achieves the minimal value on the 24th h of *L. bulgaricus* cultivation. Simultaneously, the rate of lactic acid synthesis reached the maximum value only on the 24th h of *L. bulgaricus* cultivation.

Thus, the results obtained show that 25.4 and 45.3 kDa oligochitosans significantly influence the rate of lactic acid synthesis. The presence of 1.39 × 10^−4^ mol/L oligochitosan glucosamine monomers in the culture liquid leads to a significant decrease in pH, an increase in titratable acidity, and rapid synthesis and accumulation of lactic acid compared to the control sample. Such oligochitosan activity may be explained by the mechanism of exposure to *L. bulgaricus* through extracellular, intracellular, or both extracellular and intracellular effects (58–61).

The cell wall of *L. bulgaricus* can prevent the direct binding of lactic acid to the cell membrane components and impede intracellular effects (62). Transporting molecules through the barrier of the outer layer of the rigid cell wall occurs through several subtle mechanisms or via simple diffusion (63–66). The porosity of the cell wall and the pore size determine whether oligochitosans can pass through the bacterial cell wall (67). Pore sizes vary among different bacteria and fungi, ranging from 2–4 to 8 nm (68, 69). Therefore, the radii of hydrated oligochitosans were measured using the dynamic light scattering method. According to the data obtained, the average hydrodynamic radius was 2.5 nm for 25.4 kDa oligochitosan and 3.5 nm for 45.3 kDa oligochitosan. Consequently, due to their small sizes, oligochitosans can penetrate the pores of the lactic acid bacteria cell walls and interact with plasma membrane proteins. It is likely that oligochitosans induce stress in lactic acid bacteria cells, leading to adaptive reactions that result in deviations in physiological and biochemical processes (70, 71). For instance, based on the results presented in Figure 3, the presence of 0.0025% oligochitosans in the culture liquid stimulates the acceleration of lactose catabolism, leading to increased lactic acid synthesis and accumulation.

Likely, the reason for such changes is the reaction of lactic acid bacteria stress response caused by oligochitosans. The mechanism of stress response entails the metabolic changes necessary for the survival of lactic acid bacteria (72–74). Previous studies have demonstrated that oligochitosans and chitosan, upon interacting with eukaryotic cells, induce abiotic stress, triggering physiological protective reactions (75). These protective responses may include overexpression of genes associated with carbohydrate metabolism (76), among other reactions (77–79).

At high concentrations, oligochitosans cause not only intracellular but also extracellular effects. Gram-positive bacteria have a negative charge due to the presence of phosphate groups associated with teichoic acids in the cell wall structure (80, 81). With an increase in the concentration of oligochitosans molecules, an electrostatic interaction with negatively charged lactic acid bacteria cell walls can occur (40, 82, 83). Cell walls are dynamic structures that undergo changes during replication, development, and age (84). This flexibility allows different molecules to pass through the cell wall (85). Consequently, ion immobilization of oligochitosans on the lactic acid bacteria cell wall can lead to a loss of flexibility, porosity, and alteration of its permeability (86, 87). The low permeability of the lactic acid bacteria cell walls leads to reduction of nutrient intake, resulting in a slowdown in the lactic acid synthesis and an increase in the pH of the culture liquid. This process, as depicted in Figures 1–3 and Table 1, depends on the molecular weight of oligochitosans and their concentration. Thus, two mechanisms of their action are realized depending on the concentration of oligochitosans. At low concentrations of oligochitosans, the acceleration of lactic acid synthesis occurs due to intracellular processes in *L. bulgaricus*. However, increasing the concentration of oligochitosans by four times leads to the realization of their extracellular effects, which slow down the metabolism of lactose and decrease the accumulation of lactic acid by 1.6 times at 25.4 kDa oligochitosan, or by 1.28 times at 45.3 kDa oligochitosan. The implementation of these two mechanisms of oligochitosans activity is multidirectional. As observed in Figure 2 and Table 1, the dominance of the mechanism of extracellular action of oligochitosans increases with an increase in the content of glucosamine monomers.

These observations are confirmed by the study of the effect of various concentrations of chitosan on the cultivation of *L. bulgaricus*. According to Table 1, the chitosan molecule contained 1850 glucosamine monomers and had an average hydrodynamic radius of 133.2 nm. Due to the large size of the chitosan molecules and the positively charged amino groups of glucosamine residues, this polysaccharide can interact with the cell walls of lactic acid bacteria (23). With an increase in pH and chitosan concentration, the density of its positive charge increases, and it effectively interacts with the negatively charged cell walls of *L. bulgaricus*. As can be seen from Table 1, at 0.0025% chitosan and oligochitosans, their molar concentrations differ, but the total content of glucosamine monomers remains the same (1.39 × 10^−4^ mol/L). However, at 0.0025% oligochitosans, interaction with *L. bulgaricus* leads to the realization of the intracellular mechanism of their action, which leads to an acceleration of lactic acid synthesis. At the same time, 0.0025% chitosan causes opposite effects in lactic acid bacteria, which slow down the catabolism of lactose and the lactic acid synthesis due to extracellular interaction. At 0.01% chitosan and oligochitosans, the total content of glucosamine monomers increases by 4 times and reaches 5.56 × 10^−4^ mol/L.

As a result, the total number of protonated amino groups of these polysaccharides increases, which leads to effective interaction with the lactic acid bacteria cell wall and inhibition of lactose catabolism. Moreover, the biochemical effects of oligochitosans and chitosan are unidirectional, which may indicate the same mechanism of their action due to extracellular effects. Thus, comparative studies of the effect of different concentrations of oligochitosans and chitosan on the cultivation of *L. bulgaricus* revealed two mechanisms of their action. At low concentrations, oligochitosans interact with the plasma membrane, which leads to an acceleration of metabolic processes in *L. bulgaricus*. At 0.01% oligochitosans and chitosan, they interact with the *L. bulgaricus* cell wall and affect the rate of lactic acid synthesis. This observation confirms the previously obtained results (88).

Thus, the results obtained expand the understanding of the mechanism of oligochitosans and chitosan effect on lactic acid bacteria. However, regarding fermented milk products, it is also critical to understand the oligochitosans and chitosan behavior toward lactic acid bacteria not only at cultivation but also at storage. Therefore, the next stage of the experiments represents the effect of these cationic polysaccharides on the metabolic processes of *L. bulgaricus* during long-term storage of fermented dairy products.

### 3.2 Effect of oligochitosans and chitosan on the metabolic processes of *L. bulgaricus* during long-term storage of fermented milk product

During a 17-day storage period of fermented dairy products containing oligochitosans, a slowdown in lactic acid fermentation was observed alongside a decrease in oligochitosans concentration. The interaction of oligochitosans with the plasma membrane of *Lactobacillus bulgaricus* affects intracellular processes related to lactose catabolism and cell division. Table 2 illustrates that at low concentrations of oligochitosans, the CFU value of *L. bulgaricus* were lower than in the control sample. This decrease in viable cells in the fermented dairy product led to a slowdown in the average rate of lactic acid synthesis after 17 days of storage. Consequently, there was a slight increase in LA content, maintaining low pH values compared to the control sample. At a concentration of 0.01% oligochitosans, where they interact with the *L. bulgaricus* cell wall, the synthesis of LA slows down, but a significant number of viable *L. bulgaricus* cells are preserved.

**Table 2:** Effect of OCHs and CH on pH and metabolism and content of *L*. *bulgaricus* in the fermented milk product at 17-days storing.

**Index**	**Concentration of OCHs and CH, %**				
	**0 (control)**	**0.0025**	**0.005**	**0.0075**	**0.01**
**25.4 kDa, 1% DD OCH (glucosamine monomers 140/mol OCH)**					
pH	3.80 ± 0.10^A^	3.30 ± 0.10^B^	3.66 ± 0.10^A^	3.88 ± 0.10^A^	4.35 ± 0.10^B^
LA content, mol/L	0.121 ± 0.011^A^	0,037 ± 0.001^B^	0,017 ± 0.001^C^	0,034 ± 0.002^B^	0,015 ± 0.002^C^
The average rate of LA synthesis, mol/L/h	7.3 × 10^−3*A, I*^	2.2 × 10^−3*B, I*^	1.0 × 10^−3*C, I*^	2.0 × 10^−3*B, I*^	0.9 × 10^−3*C, I*^
*L*. *bulgaricus* content, CFU/g	3.73 × 10^3A, α^	2.28 × 10^2B, α^	2.46 × 10^2B, α^	3.89 × 10^3A, α^	4.65 × 10^4C, α^
**45.3 kDa, 1.5% DD OCH (glucosamine monomers 248/mol OCH)**					
pH	3.82 ± 0.10^A^	3.48 ± 0.10^B^	3.61 ± 0.10^B^	3.91 ± 0.10^A^	3.96 ± 0.10^A^
LA content, mol/L	0.116 ± 0.01^A^	0.043 ± 0.001^B^	0.048 ± 0.001^B^	0.070 ± 0.001^C^	0.063 ± 0.001^C^
The average rate of LA synthesis, mol/L/h	6.8 × 10^−3*A, I*^	2.5 × 10^−3*B, I*^	2.8 × 10^−3*B, II*^	4.1 × 10^−3*C, II*^	3.7 × 10^−3*C, II*^
*L*. *bulgaricus* content, CFU/g	3.71 × 10^3A, α^	2.6 × 10^2B, α^	2.56 × 10^2B, β^	3.49 × 10^3A, α^	4.89 × 10^4C, α^
**350 kDa, 5% DD CH (glucosamine monomers 1850/mol CH)**					
pH	3.82 ± 0.1^A^	3.85 ± 0.1^A^	3.87 ± 0.1^A^	3.96 ± 0.1^A^	4.04 ± 0.1^A^
LA content, mol/L	0.113 ± 0.01^A^	0.102 ± 0.009^B^	0.104 ± 0.009^B^	0.072 ± 0.007^C^	0.063 ± 0.005^C^
The average rate of LA synthesis, mol/L/h	7.0 × 10^−3*A, I*^	6.0 × 10^−3*B, II*^	6.0 × 10^−3*B, III*^	4.2 × 10^−3*C, II*^	3.7 × 10^−3*C, II*^
*L*. *bulgaricus* content, CFU/g	3.69 × 10^3A, α^	3.65 × 10^3A, β^	3.59 × 10^3A, γ^	5.3 × 10^5B, β^	6.34 × 10^6C, β^

The values represent the mean ± SD (n = 3). Means without a common letter within the same column (I–III; α-γ) and row (A–C) indicate significant difference at p <0.05.

Based on the data presented in Table 2, it is evident that an increase in the concentration of chitosan leads to a reduction in the intensity of lactic acid fermentation. The most notable deceleration of lactose catabolism via homofermentative lactic acid fermentation occurred in the sample containing 0.01% chitosan. This decrease in lactose catabolism intensity in *L. bulgaricus* could be attributed to the interaction between chitosan and the bacterial cell wall. This external influence may disrupt the permeability of the cell wall to the substrate and the enzyme β-galactosidase (EC 3.2.1.23), responsible for lactose hydrolysis into glucose and galactose (89). Additionally, structural alterations in the cell wall might impede the active transport of lactose hydrolysis products into Lactobacillus cells (90). On the 17th day of storage, the *L. bulgaricus* content in the control sample of fermented milk product was measured at 3.69 × 10^3^ CFU/g. Table 2 illustrates that the inclusion of chitosan resulted in an increase in *L. bulgaricus* content, reaching 3.65 × 10^3^ CFU/g at 0.0025% chitosan, 3.59 × 10^3^ CFU/g at 0.005% chitosan, 5.3 × 10^5^ CFU/g at 0.0075% CH, and 6.34 × 10^6^ CFU/g at 0.01% chitosan. Consequently, the fermented milk product sample containing 0.01% CH contained 1,700 times more *L. bulgaricus* than the control sample.

As can be seen from Tables 1, 2, the effect of oligochitosans and chitosan on the metabolic processes of *L. bulgaricus* is multidirectional. The acceleration of metabolic processes is realized due to intracellular processes of interaction of oligochitosans with lactic acid bacteria cells. Chitosan interacting with the lactic acid bacteria cell wall slows down metabolic processes. Based on the results obtained, it is logical to assume that due to the differences in the mechanism of action of oligochitosans and chitosan, they should significantly affect the composition and content of secondary metabolites during long-term storage of fermented milk product. In this regard, the relative content of secondary metabolites in the control and experimental samples was determined using GC-MS. A typical GC-MS chromatogram of a fermented milk product extract on the 17th day of storage is shown in Supplementary Figure 1. The chromatogram contains a profile of the metabolites that were analyzed and included in Table 3. Samples of fermented dairy products with 45.3 kDa oligochitosan were chosen due to the higher values of rate of lactic acid synthesis and lactic acid content in comparison with ones with addition of 25.4 kDa oligochitosan.

**Table 3:** Relative content of the main secondary metabolites in extracts from fermented dairy products containing various concentrations of OCHs and CH on the 17th day of storage.

**Retention time**	**Substance, CAS, molecular formula**	**The relative content, %**						
		**Control**	**Concentration of 45.3 kDa OCH, %**			**Concentration of 350 kDa CH, %**		
			**0.0025**	**0.0075**	**0.01**	**0.0025**	**0.0075**	**0.01**
5.64	2-Propanol, 000067-63-0, C_3_H_8_O	8.35	19.50	2.45	1.63	4.84	2.08	n.d.
5.66	3,4-Dihydro-4,4-dimethyl-2H-1,2-benzisothiazine, 2000114-14-7, C_49_H_66_N_10_O_12_S	2.55	3.95	n.d.	n.d.	n.d.	1.69	n.d.
5.72	2-Furanmethanol, 000098-00-0, C_5_C_6_C_2_	n.d.	n.d.	n.d.	n.d.	n.d.	2.48	2.05
9.03	3,5-dimethyl-4-deuteroxymethyl-isoxazole, 2000024-72-1, C_12_H_12_N_2_O	n.d.	n.d.	n.d.	n.d.	n.d.	n.d.	2.77
9.90	4H-Pyran-4-one, 3-hydro-xy-2-methyl, 000118-71-8, C_6_H_6_O_3_	10.17	0.91	1.21	1.22	0.99	2.35	8.85
10.33	Carbamic acid, butyl-, ethyl ester, 000591-62-8, C_7_H_15_NO_2_	4.13	n.d.	n.d.	n.d.	n.d.	n.d.	n.d.
10.46	2,3-Dihydro-3,5-dihydroxy-6-methyl-4H-pyran-4-one, 028564-83-2, C_6_H_8_O_4_	37.86	26.95	44.44	37.24	37.90	35.23	20.70
10.67	Benzoic acid, 000065-85-0, C_7_H_6_O_2_	8.87	9.22	7.60	8.68	12.40	12.77	9.62
13.25	Pentadecanoic acid, 001002-84-2, C_15_H_30_O_2_	n.d.	n.d.	n.d.	n.d.	n.d.	1.31	1.28
19.60	Hexadecanoic acid, methyl ester, 000112-39-0, C_17_H_34_O_2_	0.89	0.69	0.73	1.26	0.60	1.14	0.90
19.95	n-Hexadecanoic acid, 000057-10-3, C_16_H_32_O_2_	n.d.	n.d.	n.d.	n.d.	n.d.	n.d.	7.91
21.28	8-Octadecenoic acid, methyl ester, (E)-, 026528-50-7, C_19_H_36_O_2_	0.66	n.d.	n.d.	n.d.	n.d.	n.d.	n.d.
21.29	12-Octadecenoic acid, methyl ester, 056554-46-2, C_19_H_36_O_2_	0.63	n.d.	n.d.	n.d.	n.d.	n.d.	n.d.
21.39	Methyl stearate, 000112-61-8, C_19_H_38_O_2_	0.44	0.37	n.d.	n.d.	0.37	n.d.	n.d.
21.49	Heptadecanoic acid, 16-methyl-, methyl ester, 005129-61-3, C_19_H_38_O_2_	0.49	n.d.	0.39	n.d.	n.d.	0.74	0.55
21.64	Cis-11-octadecenoic acid, 000506-17-2, C_18_H_34_O_2_	n.d.	n.d.	n.d.	n.d.	n.d.	5.79	21.58
21.82	Octadecanoic acid, 000057-11-4, C_18_H_36_O_2_	n.d.	n.d.	n.d.	n.d.	1.07	2.09	2.93
29.91	Cholest-5-en-3-ol, 2000683-53-6, C_27_H_46_O	1.34	0.98	1.22	2.67	1.25	2.47	1.35

The analysis of the results shows that 0.0025% of 45.3 kDa oligochitosan accelerates the synthesis of propanol, leading to a 230% increase in its relative content compared to the control sample. Propanol metabolism is closely related to the synthesis of lactic acid (91), the content of which was 260% higher in this sample than in the control sample. The acceleration of lactic acid synthesis was accompanied by an increase in propanol synthesis, consistent with previously obtained results (92, 93). With an increase in oligochitosan concentration, the process of lactic acid synthesis slows down and the relative propanol content decreases. Another important component of secondary metabolites presented in Table 3 is benzoic acid. It was revealed, that different concentrations of oligochitosan had practically no effect on its synthesis compared to the control sample.

Fermented dairy products also contained a significant number of phenolic compounds: 3,4-dihydro-4,4-dimethyl-2H-1,2-benzisothiazine; 2-furanmethanol; 3,5-dimethyl-4-deuteroxymethyl-isoxazole; 4H-pyran-4-one, 3-hydroxy-2-methyl; 2,3-dihydro-3,5-dihydroxy-6-methyl-4H-pyran-4-one. Their total content in the control sample reached 54.68%. In the experimental samples of fermented dairy products with oligochitosan, the content of phenolic compounds ranged from 31.81% at a concentration of 0.0025% 45.3 kDa oligochitosans to 45.65% at a concentration of 0.005% 45.3 kDa oligochitosans. It is important to note that phenolic substances exhibit antioxidant properties and affect the sensory characteristics of fermented dairy products (94, 95).

Fatty acids have a significant role in shaping the sensory attributes of fermented dairy products. Saturated fatty acids made up 1.81% of the control sample, including methyl stearate (0.44%), heptadecanoic acid, methyl ester (0.89%), and heptadecanoic acid, 16-methyl-, and methyl ester (0.49%). Furthermore, the control sample had two unsaturated fatty acids, 8-octadecanoic acid, methyl ester, (E)—(0.66%) and 12-octadecenoic acid, methyl ester (0.63%), that were absent from the other samples. The control sample of fermented dairy product had a total fatty acid level of 3.11%.

Variations in the content of 45.3 kDa oligochitosan led to notable alterations in the fatty acid synthesis in fermented dairy products. Table 3 shows that samples with an oligochitosan of 45.3 kDa were devoid of unsaturated fatty acids. Heptadecanoic acid, methyl ester (C_17_H_34_O_2_), methyl stearate (C_19_H_38_O_2_), and hepta-decanoic acid, 16-methyl-, methyl ester (C_19_H_38_O_2_) are examples of saturated fatty acids. Their overall content was lower than that of the control sample (3.11%), although it did grow significantly with increasing OCH concentration, reaching 1.06% at 0.0025% oligochitosan, 1.15% at 0.0075% oligochitosan, and 1.26% at 0.01% oligochitosan. Saturated fatty acids have been demonstrated in the past to be a crucial growth factor for several lactic acid bacteria (9, 96).

The main and secondary metabolite composition of the fermented milk product underwent considerable alterations as a result of the 45.3 kDa oligochitosan, according to the data shown in Tables 1–3. This is because the 45.3 kDa oligochitosan's intracellular mechanism of action causes biochemical alterations, which are responsible for the fermented milk product's low pH, high propanol and LA content, minimal saturated fatty acid content, and low *L. bulgaricus* content. Consequently, the consumer qualities of the fermented dairy product with 45.3 kDa oligochitosan are lost. Nevertheless, in light of the effects that have been found, oligochitosans may be utilized as a stimulant for lactic acid synthesis during lactic acid bacteria culture.

Unlike the 45.3 kDa oligochitosan, chitosan's effect on *L. bulgaricus* occurs via extracellular interaction. According to Tables 2, 3, with an increase in the chitosan concentration, the lactic acid synthesis slows down and the synthesis of propanol is prevented. Fermented dairy product containing 0.0025 and 0.0075% has a 140% higher content of benzoic acid relative to the control sample. It is well-known that a relatively high content of benzoic acid can prevent contamination of fermented milk products by yeast and other microorganisms (97–99). Table 3 shows that chitosan causes additional synthesis of some saturated fatty acids: pentadecanoic acid; hexadecanoic acid, methyl ester; n-hexadecanoic acid; methyl stearate; heptadecanoic acid, 16-methyl-, methyl ester; octadecanoic acid; as well as one unsaturated fatty acid—cis-11-octadecenoic acid. At 0.0025% chitosan, fermented milk product contained 2.04% saturated fatty acids and did not contain unsaturated acids. However, 0.0075% chitosan caused an increase in the content of saturated fatty acids to 5.28% and unsaturated by 5.79%. Fermented milk product with 0.01% chitosan had the highest content of fatty acids after 17 days of storage: 13.57% saturated fatty acids and 21.58% unsaturated fatty acid (cis-11-octadecanoic acid). As presented in Table 2, with an increase in the chitosan concentration, the content of *L. bulgaricus* increased as well. At the same time, according to Table 3, there was a significant increase in the content of fatty acids, which confirms their importance for *L. bulgaricus* growth. Thus, chitosan in interaction with *L. bulgaricus* causes metabolic shifts in the synthesis of secondary metabolites. As a result, lactic acid bacteria produce antifungal metabolites such as organic acids, phenolic compounds and a wide range of carboxylic acids and their esters (100, 101). These substances determine the high sensory characteristics of fermented milk product (102).

Furthermore, the shelf life of a fermented dairy product is a crucial indicator. Titratable acidity is seen to rise with an increase in storage duration. The titratable acidity of fermented dairy products, which should not be more than 140°T (49), determines their maximum shelf life. This value was attained in the control sample on the 5th day of storage and surpassed by 145% on the 17th day of storage (Table 4).

**Table 4:** Effect of 350 kDa CH on titratable acidity of fermented milk product during storage.

**Storage time, days**	**Titrated acidity**, **°T**				
	**Concentration of 350 kDa CH, %**				
	**0 (control)**	**0.0025**	**0.005**	**0.0075**	**0.01**
1	90 ± 3^A, I^	89 ± 2^A, I^	82 ± 2^B, I^	83 ± 1^B, I^	75 ± 1^C, I^
3	117 ± 2^A, II^	110 ± 3^B, II^	106 ± 1^B, II^	104 ± 3^B, C, II^	100 ± 4^C, II^
5	*140 ± 4* ^A, III^	132 ± 3^B, III^	118 ± 3^C, III^	110 ± 2^D, II, III^	103 ± 2^E, II^
7	157 ± 3^A, IV^	*145 ± 2* ^B, IV^	132 ± 3^C, IV^	116 ± 4^D, III^	114 ± 1^D, III^
9	180 ± 2^A, V^	164 ± 1^B, V^	*148 ± 3* ^C, V^	128 ± 4^D, IV^	126 ± 4^D, IV^
11	185 ± 4^A, V, VI^	170 ± 3^B, VI^	155 ± 2^D, VI^	138 ± 2^D, V^	129 ± 4^E, IV, V^
13	192 ± 5^A, VI, VII^	179 ± 2^B, VII^	161 ± 4^C, VI, VII^	142 ± 5^D, V, VI^	131 ± 3^E, IV, V^
15	198 ± 4^A, VII, VIII^	185 ± 4^B, VII, VIII^	164 ± 2^C, VII^	*145 ± 3* ^D, VI^	132 ± 4^E, IV, V^
17	203 ± 2^A, VIII^	191 ± 5^B, VIII^	186 ± 2^B, VIII^	155 ± 3^C, VII^	*138 ± 4* ^D, V^

The values represent the mean ± SD (n = 3). Means without a common letter within the same column (I–VIII) and row (A–E) indicate significant difference at p <0.05.

Experimental samples containing 0.0025, 0.005, 0.0075, and 0.01% chitosan reached the required titratable acidity levels on the 7, 9, 13, and 17th days of storage, respectively. Surprisingly, the optimal concentration of 0.01% chitosan maintained the titratable acidity of the fermented dairy product at 138°T on 17th day of storage. In general, this concentration increased the shelf life of the fermented dairy product by three times compared to the control sample. Additionally, lactic acid fermentation process was slowed down, while maintaining a high content of lactic acid bacteria and a low concentration of lactic acid.

An important sensory indicator of fermented milk product during storage is the clot density of the clot (103, 104). On the 17th day of storage, the viscosity of the control sample was 168 MPa × s, while the viscosity of the sample with 0.01% chitosan reached to 176 MPa × s. Thus, in the presence of chitosan, the liquefaction of the clot slowed down during storage. This is attributed to the fact that the experimental sample contained less lactic acid and maintained a relatively high pH value. Consequently, hydrolysis of milk proteins and destruction of casein micelles does not occur in experimental samples, as confirmed by SDS-electrophoretic examination (Supplementary Figure 2).

**Figure 4:**
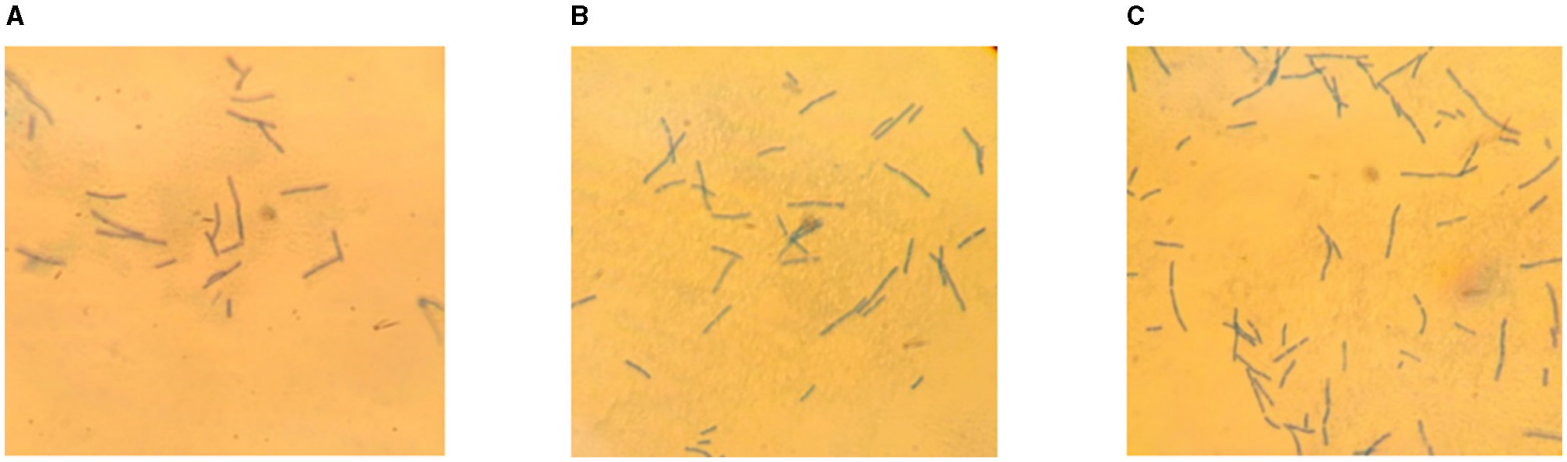
Microscopic preparations of fermented milk product: control sample **(A)**, 0.0075% CH **(B)**, 0.01% CH **(C)**.

Further, in order to clarify the effect of chitosan on the form of LAB and confirm the absence of extraneous microflora in the samples, fixed preparations were obtained and studied after 17 days of storage. The results of microscopic studies are depicted in Figure 4.

Analysis of the microscopic preparations showed that interaction of *L. bulgaricus* with chitosan at concentrations of 0.0075 and 0.01% did not lead to a change in their rod shape. There were no foreign microflora in the samples examined. This may be due to the high content of secondary metabolites, such as benzoic acid, which prevent contamination of the product by other microorganisms (105).

Thus, the effective interaction of *L. bulgaricus* with chitosan is due to the presence in its 1850/mol structure of glucosamine monomers which, in an acidic environment, perform a multipoint ionic interaction with negatively charged teichoic acid molecules of the lactic acid bacteria cell wall. With increasing chitosan concentration, the process of lactose catabolism by *L. bulgaricus* and the accumulation of the lactic acid slows down. As a result of this process, the shelf life of the fermented milk product is significantly increased. At the same time 0.01% chitosan provided a high level of lactic acid bacteria and improved sensory characteristics of the product during 17 days storage. The concentrations of chitosan used did not give astringent taste to the fermented dairy product.

## 4 Conclusions

This study presents the results of a comparative study of the effect of oligochitosans and chitosan on the lactic acid fermentation process during cultivation of *L. bulgaricus* and long-term storage. The foundation of this study was the potential utilization of the ionic interaction between cationic polysaccharides and negatively charged *L. bulgaricus* cells to regulate lactic acid synthesis. Depending on the molecular weight, concentration, and molecules size of oligochitosans and chitosan effect on lactic acid synthesis due to intracellular or extracellular interactions during cultivation of *L. bulgaricus* and long-term storage of the fermented dairy product. When *L. bulgaricus* was cultured in the presence of 0.0025% 25.4 kDa oligochitosan, 45.3 kDa oligochitosan, and 350 kDa chitosan, their molar concentrations were 0.99 × 10^−6^, 0.56 × 10^−6^, and 0.75 × 10^−7^ mol/L, respectively. At the same time, the total content of glucosamine monomers in these samples was the same and amounted to 1.39 × 10^−4^ mol/L. After 24 h of cultivation, the lactic acid content in the fermented milk samples was 0.087 mol/L in the control sample, 0.255 mol/L in sample with 25.4 kDa oligochitosan, 0.228 mol/L in sample with 45.3 kDa oligochitosan, and 0.089 mol/L in sample with 350 kDa chitosan. At equal glucosamine monomers content, oligochitosans accelerate the synthesis of lactic acid, while chitosan has no such an effect compared to the control sample. These results indicate different mechanisms of action of oligochitosans and chitosan on the synthesis of lactic acid. With increasing concentrations of oligochitosans, a slowdown in the synthesis of lactic acid is observed. At the same time, its content remained higher than in the control sample. During long-term storage (17 days) of dairy product fermented with oligochitosan, further accumulation of lactic acid, decrease in pH and *L. bulgaricus* content occurred. The results obtained confirm that oligochitosans can be used as stimulators of lactic acid synthesis based on lactose containing substrates industrial fermentation using *L. bulgaricus* starter cultures.

Chitosan with molecular weight 350 kDa had the opposite effect on metabolic processes in fermented dairy product. With increasing chitosan concentration, lactic acid and propanol synthesis slowed down and the content of saturated and unsaturated fatty acids significantly increased. As a result, the titratable acidity decreased and the *L. bulgaricus* content increased. The result was an increase in the shelf life of the fermented dairy product. Chitosan provided low acidity of the product, high content of biologically active substances, increased *L. bulgaricus* content and better sensory characteristics compared to samples with addition of oligochitosans. The concentrations of chitosan used did not give astringent taste to the fermented dairy product. Further prospects are related to the study of the effect of 350 kDa chitosan on the technological processes of lactic acid bacteria various strains cultivation used in the production of commercial fermented dairy products.

## Data Availability

The original contributions presented in the study are included in the article/Supplementary material, further inquiries can be directed to the corresponding authors.

## Supplementary material

Supplementary Figure 1 A typical GC-MS chromatogram of an extract from fermented dairy product on the 17th day of storage.

Supplementary Figure 2 SDS-electrophoregram of proteins of fermented dairy product on the 17th day of storage: control sample (5), 0.01% CH (4), 0.0075% CH (3), 0.0025% CH (2), original milk (1).

## References

[1] Ambikapathi R Schneider KR Davis B Herrero M Winters P Fanzo JC. Global food systems transitions have enabled affordable diets but had less favourable outcomes for nutrition, environmental health, inclusion and equity. Nat Food. (2022) 3:764–79. 10.1038/s43016-022-00588-7 37118149

[2] Granato D Barba FJ Bursać Kovacević D Lorenzo JM Cruz AG Putnik P. Functional foods: product development, technological trends, efficacy testing, and safety. Food Sci Technol. (2020) 11:93–118. 10.1146/annurev-food-032519-051708 31905019

[3] Marco ML Sanders ME Gänzle M Arrieta MC Cotter PD De Vuyst L. The International Scientific Association for Probiotics and Prebiotics (ISAPP) consensus statement on fermented foods. Nat Rev Gastroenterol Hepatol. (2021) 18:196–208. 10.1038/s41575-020-00390-5 33398112 PMC7925329

[4] Abdullah Thaidi NI Rios-Solis L Halim M. Fermented milk: the most famous probiotic, prebiotic, and synbiotic food carrier. Probiot Prebiot Foods. (2021) 4:135–51. 10.1016/B978-0-12-819662-5.00012-4

[5] Ahansaz N Tarrah A Pakroo S Corich V Giacomini A. Lactic acid bacteria in dairy foods: prime sources of antimicrobial compounds. Fermentation. (2023) 9:964. 10.3390/fermentation9110964

[6] Raman J Kim JS Choi KR Eun H Yang D Ko YJ. Application of lactic acid bacteria (LAB) in sustainable agriculture: advantages and limitations. Int J Mol Sci. (2022) 23:7784. 10.3390/ijms23147784 35887142 PMC9322495

[7] Arsov A Ivanov I Tsigoriyna L Petrov K Petrova P. *In vitro* production of galactooligosaccharides by a novel β-galactosidase of *Lactobacillus bulgaricus*. Int J Mol Sci. (2022) 23:14308. 10.3390/ijms232214308 36430784 PMC9697242

[8] Abbasalizadeh S Hejazi MA Pesaran Hajiabbas M. Kinetics of β-galactosidase production by *Lactobacillus bulgaricus* during pH controlled batch fermentation in three commercial bulk starter media. Appl Food Biotechnol. (2015) 2:39–47. 10.22037/afb.v2i4.951229708228

[9] Wang Y Wu J Lv M Shao Z Hungwe M Wang J. Metabolism characteristics of lactic acid bacteria and the expanding applications in food industry. Front Bioeng Biotechnol. (2021) 9:612285. 10.3389/fbioe.2021.612285 34055755 PMC8149962

[10] Ashaolu TJ. Safety and quality of bacterially fermented functional foods and beverages: a mini review. Food Qual Saf. (2020) 4:123–7. 10.1093/fqsafe/fyaa003

[11] Ntsefong GN Lodygin A Evdokimov I Oboturova N Rzhepakovsky I Nersesyan T. Polymer selection for microencapsulation of probiotics: impact on viability, stability, and delivery in functional foods for improved manufacturing and product development in the food industry. Potr S J F Sci. (2023) 17:712–7. 10.5219/1902

[12] Hussien H Abd-Rabou HS Saad MA. The impact of incorporating *Lactobacillus acidophilus* bacteriocin with inulin and FOS on yogurt quality. Sci Rep. (2022) 12:13401. 10.1038/s41598-022-17633-x 35927320 PMC9352778

[13] Sreekumar S Wattjes J Niehues A Mengoni T Mendes AC Morris ER. Biotechnologically produced chitosans with nonrandom acetylation patterns differ from conventional chitosans in properties and activities. Nat Commun. 13:7125 (2022). 10.1038/s41467-022-34483-3 36418307 PMC9684148

[14] Zhang W Sadeghi A Karaca AC Zhang J Jafari SM. Carbohydrate polymer-based carriers for colon targeted delivery of probiotics. Crit Rev Food Sci Nutr. (2023) 2023:2257321. 10.1080/10408398.2023.2257321 37702799

[15] Castro-Muñoz R Kharazmi MS Jafari SM. Chitosan-based electrospun nanofibers for encapsulating food bioactive ingredients: a review. Int J Biol Macromol. (2023) 245:125424. 10.1016/j.ijbiomac.2023.125424 37343613

[16] Hadidi M Pouramin S Adinepour F Haghani S Jafari SM. Chitosan nanoparticles loaded with clove essential oil: characterization, antioxidant and antibacterial activities. Carbohydr Polym. (2020) 236:116075. 10.1016/j.carbpol.2020.116075 32172888

[17] Akbari-Alavijeh S Shaddel R Jafari SM. Encapsulation of food bioactives and nutraceuticals by various chitosan-based nanocarriers. Food Hydrocolloids. (2020) 105:105774. 10.1016/j.foodhyd.2020.10577437343613

[18] Rehman K Hollah C Wiesotzki K Heinz V Aganovic K Rehman R. Insect-derived chitin and chitosan: a still unexploited resource for the edible insect sector. Sustainability. (2023) 15:4864. 10.3390/su15064864

[19] Jiménez-Gómez CP Cecilia JA. Chitosan: a natural biopolymer with a wide and varied range of applications. Molecules. (2020) 25:3981. 10.3390/molecules25173981 32882899 PMC7504732

[20] Wang H Roman M. Effects of chitosan molecular weight and degree of deacetylation on chitosan–cellulose nanocrystal complexes and their formation. Molecules. (2023) 28:1361. 10.3390/molecules28031361 36771029 PMC9920826

[21] Piekarska K Sikora M Owczarek M Józwik-Pruska J Wiśniewska-Wrona M. Chitin and chitosan as polymers of the future-obtaining, modification, life cycle assessment and main directions of application. Polymers. (2023) 15:793. 10.3390/polym15040793 36850077 PMC9959150

[22] Román-Doval R Torres-Arellanes SP Tenorio-Barajas AY Gómez-Sánchez A Valencia-Lazcano AA. Chitosan: properties and its application in agriculture in context of molecular weight. Polymers. (2023) 15:2867. 10.3390/polym15132867 37447512 PMC10346603

[23] Harugade A Sherje AP Pethe A. Chitosan: a review on properties, biological activities and recent progress in biomedical applications. React Funct Polym. (2023) 191:105634. 10.1016/j.reactfunctpolym.2023.105634

[24] Ang LF Por LY Yam MF. Study on different molecular weights of chitosan as an immobilization matrix for a glucose biosensor. PLoS ONE. (2013) 8:e70597. 10.1371/journal.pone.0070597 23940599 PMC3734260

[25] Gonçalves C Ferreira N Lourenço L. Production of low molecular weight chitosan and chitooligosaccharides (COS): a review. Polymers. (2021) 13:2466. 10.3390/polym13152466 34372068 PMC8348454

[26] Zhang W Hadidi M Karaca AC Hedayati S Tarahi M Assadpour E. Chitosan-grafted phenolic acids as an efficient biopolymer for food packaging films/coatings. Carbohydr Polym. (2023) 314:120901. 10.1016/j.carbpol.2023.120901 37173040

[27] Chou CM Mi FL Horng JL Lin LY Tsai ML Liu CL. Characterization and toxicology evaluation of low molecular weight chitosan on zebrafish. Carbohydr Polym. (2020) 240:116164. 10.1016/j.carbpol.2020.116164 32475540

[28] Al-Jbour ND Beg MDH Gimbun J Alam AKMM. Preparation and characterization of low molecular weight chitosan with different degrees of deacetylation by the acid hydrolysis method. Int J App Pharm. (2021) 2021:153–64. 10.22159/ijap.2021v13i2.32229

[29] Villegas-Peralta Y López-Cervantes J Madera Santana TJ Sánchez-Duarte RG Sánchez-Machado DI del Rosario Martínez-Macías M. Impact of the molecular weight on the size of chitosan nanoparticles: characterization and its solid-state application. Polym Bull. (2021) 78:813–32. 10.1007/s00289-020-03139-x

[30] Boamah PO Onumah J Agolisi MH Idan F. Application of low molecular weight chitosan in animal nutrition, husbandry, and health: a review. Carbohydr Polym Technol Appl. (2023) 6:100329. 10.1016/j.carpta.2023.100329

[31] Liu Y Yuan Y Duan S Li C Hu B Liu A. Preparation and characterization of chitosan films with three kinds of molecular weight for food packaging. Int J Biol Macromol. (2020) 155:249–59. 10.1016/j.ijbiomac.2020.03.217 32234438

[32] Kaczmarek MB Struszczyk-Swita K Li X Szczesna-Antczak M Daroch M. Enzymatic modifications of chitin, chitosan, and chitooligosaccharides. Front Bioeng Biotechnol. (2019) 7:243. 10.3389/fbioe.2019.00243 31612131 PMC6776590

[33] Zou X Yang J Wang Y Li H Yu Y Zhang G. Advances in characterization and biological activities of chitosan and chitosan oligosaccharides. Food Chem. (2016) 190:1174–81. 10.1016/j.foodchem.2015.06.076 26213092

[34] Kulikov S Tikhonov V Blagodatskikh I Bezrodnykh E Lopatin S Khairullin R. Molecular weight and pH aspects of the efficacy of oligochitosan against methicillin-resistant *Staphylococcus aureus* (MRSA). Carbohydr Polym. (2012) 87:545–50. 10.1016/j.carbpol.2011.08.017 34663002

[35] Muanprasat C Chatsudthipong V. Chitosan oligosaccharide: biological activities and potential therapeutic applications. Pharmacol Therapeut. (2017) 170:80–97. 10.1016/j.pharmthera.2016.10.013 27773783

[36] Guan G Azad MAK Lin Y Kim SW Tian Y Liu G. Biological effects and applications of chitosan and chito-oligosaccharides. Front Physiol. (2019) 10:516. 10.3389/fphys.2019.00516 31133871 PMC6514239

[37] Meng Q Sun Y Cong H Hu H Xu FJ. An overview of chitosan and its application in infectious diseases. Drug Deliv Transl Res. (2021) 11:1340–51. 10.1007/s13346-021-00913-w 33496926 PMC7837079

[38] Teixeira-Santos R Lima M Gomes LC Mergulhão FJ. Antimicrobial coatings based on chitosan to prevent implant-associated infections: a systematic review. iScience. (2021) 24:103480. 10.1016/j.isci.2021.103480 34927024 PMC8652012

[39] Roy S Rhim JW. Fabrication of chitosan-based functional nanocomposite films: effect of quercetin-loaded chitosan nanoparticles. Food Hydrocolloids. (2021) 121:107065. 10.1016/j.foodhyd.2021.107065

[40] Ristić T Lasič S Kosalec I Bračič M Fras-Zemljič L. The effect of chitosan nanoparticles onto Lactobacillus cells. React Funct Polym. (2015) 97:56–62. 10.1016/j.reactfunctpolym.2015.10.00734082927

[41] Riaz Rajoka MS Mehwish HM Wu Y Zhao L Arfat Y Majeed K. Chitin/chitosan derivatives and their interactions with microorganisms: a comprehensive review and future perspectives. Crit Rev Biotechnol. (2020) 40:365–79. 10.1080/07388551.2020.1713719 31948287

[42] Kovács R Erdélyi L Fenyvesi F Balla N Kovács F Vámosi G. Concentration-dependent antibacterial activity of chitosan on *Lactobacillus plantarum*. Pharmaceutics. (2023) 15:18. 10.3390/pharmaceutics15010018 36678647 PMC9862870

[43] Ebrahimnejad P Khavarpour M Khalili S. Survival of *Lactobacillus acidophilus* as probiotic bacteria using chitosan nanoparticles. IJE. (2017) 30:456–63. 10.5829/idosi.ije.2017.30.04a.01

[44] Bodnár M Fazekas E Nagy T Miltner N Kalló G Kerekes K. Synthesis of galacto-oligosaccharides in milk by using *Bifidobacterium bifidum* β-galactosidases (Saphera 2600L and Nola Fit 5500) immobilized on chitosan beads. Food Bioprocess Technol. (2023). 10.1007/s11947-023-03222-x

[45] Shramko MI Lodygin AD Evdokimov IA Sushinskaya NV Kurchenko VP. Influence of oligochitosans and highly molecular chitosan on *Lactobacillus bulgaricus* cultivation. IOP Conf Ser Earth Environ Sci. (2020) 548:e082066. 10.1088/1755-1315/548/8/082066

[46] Bajrami D Fischer S Barth H Hossain SI Cioffi N Mizaikoff B. Antimicrobial efficiency of chitosan and its methylated derivative against *Lentilactobacillus parabuchneri* biofilms. Molecules. (2022) 27:8647. 10.3390/molecules27248647 36557784 PMC9786053

[47] Berezin BB Bezrodnykh EA Blagodatskikh IV Yamskov IA Tikhonov VE. Extraction of residual heavy metals from commercial chitosan and approach to oligochitosan hydrochloride. Carbohydr Polym. (2019) 215:316–21. 10.1016/j.carbpol.2019.03.072 30981360

[48] Marković B Ignjatović J Vujadinović M Savić V Vladimirov S Karljiković-Rajić K. Inter-laboratory verification of European pharmacopoeia monograph on derivative spectrophotometry method and its application for chitosan hydrochloride. Spectrochim Acta A Mol Biomol Spectr. (2015) 150:792–8. 10.1016/j.saa.2015.06.022 26112102

[49] Tomovska J Gjorgievski N Makarijoski B. Examination of pH, titratable acidity and antioxidant activity in fermented milk. JMSE-A. (2016) 6:326–33. 10.17265/2161-6213/2016.11-12.006

[50] Kurchenko V Halavach T Tikhonov V Shramko M Alieva L. Patterns of changes in the properties of a fermented milk drink with *Lactobacillus acidophilus* from the molecular weights of chitosans. Intell Biotechnol Nat Synthet Biolog Act Substances. (2023) 2931:e050008. 10.1063/5.0177862

[51] Jovanovic S Barac M Macej O Vucic T Lacnjevac C. SDS-PAGE analysis of soluble proteins in reconstituted milk exposed to different heat treatments. Sensors. (2007) 7:371–83. 10.3390/s7030371

[52] Blagodatskikh IV Kulikov SN Vyshivannaya OV Bezrodnykh EA Tikhonov VE. N-reacetylated oligochitosan: pH dependence of self-assembly properties and antibacterial activity. Biomacromolecules. (2017) 18:1491–8. 10.1021/acs.biomac.7b00039 28375595

[53] Saxena D Maitra R Bormon R Czekanska M Meiers J Titz A. Tackling the outer membrane: facilitating compound entry into Gram-negative bacterial pathogens. NPJ Antimicrob Resist. (2023) 1:17. 10.1038/s44259-023-00016-1 PMC11721184 39843585

[54] Yan D Li Y Liu Y Li N Zhang X Yan C. Antimicrobial properties of chitosan and chitosan derivatives in the treatment of enteric infections. Molecules. (2021) 26:7136. 10.3390/molecules26237136 34885715 PMC8659174

[55] Yang S Bai M Kwok LY Zhong Z Sun Z. The intricate symbiotic relationship between lactic acid bacterial starters in the milk fermentation ecosystem. Crit Rev Food Sci Nutr. (2023) 2023:1–18. 10.1080/10408398.2023.2280706 37983125

[56] Desai N Rana D Salave S Gupta R Patel P Karunakaran B. Chitosan: a potential biopolymer in drug delivery and biomedical applications. Pharmaceutics. (2023) 15:1313. 10.3390/pharmaceutics15041313 37111795 PMC10144389

[57] Savard T Beaulieu C Boucher I Champagne CP. Antimicrobial action of hydrolyzed chitosan against spoilage yeasts and lactic acid bacteria of fermented vegetables. J Food Protect. (2002) 65:828–33. 10.4315/0362-028X-65.5.828 12030295

[58] Varlamov VP Mysyakina IS. Chitosan in biology, microbiology, medicine, and agriculture. Microbiology. (2018) 87:712–5. 10.1134/S0026261718050168

[59] Kravanja G PrimoŽič M Knez Ž Leitgeb M. Chitosan-based (nano)materials for novel biomedical applications. Molecules. (2019) 24:1960. 10.3390/molecules24101960 31117310 PMC6572373

[60] Hosseinnejad M Jafari SM. Evaluation of different factors affecting antimicrobial properties of chitosan. Int J Biol Macromol. (2016) 85:467–75. 10.1016/j.ijbiomac.2016.01.022 26780706

[61] Ke CL Deng FS Chuang CY Lin CH. Antimicrobial actions and applications of chitosan. Polymers. (2021) 13:904. 10.3390/polym13060904 33804268 PMC7998239

[62] Stirke A Celiesiute-Germaniene R Zimkus A Zurauskiene N Simonis P Dervinis A. The link between yeast cell wall porosity and plasma membrane permeability after PEF treatment. Sci Rep. (2019) 9:1–10. 10.1038/s41598-019-51184-y 31611587 PMC6791849

[63] Bajaj H Acosta Gutierrez S Bodrenko I Malloci G Scorciapino MA Winterhalter M. Bacterial outer membrane porins as electrostatic nanosieves: exploring transport rules of small polar molecules. ACS Nano. (2017) 11:5465–73. 10.1021/acsnano.6b08613 28485920

[64] Özkan M Yilmaz H Ergenekon P Erdogan EM Erbakan M. Microbial membrane transport proteins and their biotechnological applications. World J Microbiol Biotechnol. (2024) 40:71. 10.1007/s11274-024-03891-6 38225445 PMC10789880

[65] Dyett BP Yu H Strachan J Drummond CJ Conn CE. Fusion dynamics of cubosome nanocarriers with model cell membranes. Nat Commun. (2019) 10:4492. 10.1038/s41467-019-12508-8 31582802 PMC6776645

[66] Silale A Zhu Y Witwinowski J Smith RE Newman KE Bhamidimarri SP. Dual function of OmpM as outer membrane tether and nutrient uptake channel in diderm Firmicutes. Nat Commun. (2023) 14:7152. 10.1038/s41467-023-42601-y 37932269 PMC10628300

[67] Li ZP Paterlini A Glavier M Bayer EM. Intercellular trafficking via plasmodesmata: molecular layers of complexity. Cell Mol Life Sci. (2021) 78:799–816. 10.1007/s00018-020-03622-8 32920696 PMC7897608

[68] Rojewska M Smułek W Kaczorek E Prochaska K. Langmuir monolayer techniques for the investigation of model bacterial membranes and antibiotic biodegradation mechanisms. Membranes. (2021) 11:707. 10.3390/membranes11090707 34564524 PMC8471293

[69] Stater EP Sonay AY Hart C Grimm J. The ancillary effects of nanoparticles and their implications for nanomedicine. Nat Nanotechnol. (2021) 16:1180–94. 10.1038/s41565-021-01017-9 34759355 PMC9031277

[70] Imlay J. The molecular mechanisms and physiological consequences of oxidative stress: lessons from a model bacterium. Nat Rev Microbiol. (2013) 11:443–54. 10.1038/nrmicro3032 23712352 PMC4018742

[71] Mukarram M Khan MMA Kurjak D Corpas FJ. Chitosan oligomers (COS) trigger a coordinated biochemical response of lemongrass (*Cymbopogon flexuosus*) plants to palliate salinity-induced oxidative stress. Sci Rep. (2023) 13:8636. 10.1038/s41598-023-35931-w 37244976 PMC10224966

[72] Olicón-Hernández DR Uribe-Alvarez C Uribe-Carvajal S Pardo JP Guerra-Sánchez G. Response of ustilago maydis against the stress caused by three polycationic chitin derivatives. Molecules. (2017) 22:1745. 10.3390/molecules22121745 29215563 PMC6149792

[73] Hosseini Nezhad M Hussain MA Britz ML. Stress responses in probiotic *Lactobacillus casei*. Crit Rev Food Sci Nutr. (2014) 55:740–9. 10.1080/10408398.2012.675601 24915363

[74] Parlindungan E May BK Jones OAH. Metabolic insights into the effects of nutrient stress on *Lactobacillus plantarum* B21. Front Mol Biosci. (2019) 6:75. 10.3389/fmolb.2019.00075 31544106 PMC6730488

[75] Thambiliyagodage C Jayanetti M Mendis A Ekanayake G Liyanaarachchi H Vigneswaran S. Recent advances in chitosan-based applications-a review. Materials. (2023) 16:2073. 10.3390/ma16052073 36903188 PMC10004736

[76] Ricciardi A Castiglione Morelli MA Ianniello RG Parente E Zotta T. Metabolic profiling and stress response of anaerobic and respiratory cultures of *Lactobacillus plantarum* C17 grown in a chemically defined medium. Ann Microbiol. (2015) 65:1639–48. 10.1007/s13213-014-1003-z

[77] Adam C Paolini L Gueguen N Mabilleau G Preisser L Blanchard S. Acetoacetate protects macrophages from lactic acidosis-induced mitochondrial dysfunction by metabolic reprograming. Nat Commun. (2021) 12:7115. 10.1038/s41467-021-27426-x 34880237 PMC8655019

[78] Papadimitriou K Alegría Á Bron PA de Angelis M Gobbetti M Kleerebezem M. Stress physiology of lactic acid bacteria. Microbiol Mol Biol Rev. (2016) 80:837–90. 10.1128/MMBR.00076-15 27466284 PMC4981675

[79] Yang H He M Wu C. Cross protection of lactic acid bacteria during environmental stresses: stress responses and underlying mechanisms. LWT. (2021) 144:111203. 10.1016/j.lwt.2021.111203

[80] Pasquina-Lemonche L Burns J Turner RD Kumar S Tank R Mullin N. The architecture of the gram-positive bacterial cell wall. Nature. (2020) 582:294–7. 10.1038/s41586-020-2236-6 32523118 PMC7308169

[81] Rohde M. The gram-positive bacterial cell wall. Microbiol Spectr. (2019) 7:3. 10.1128/microbiolspec.GPP3-0044-2018 31124431 PMC11086966

[82] Yu L O'Sullivan DJ. Immobilization of whole cells of *Lactococcus lactis* containing high levels of a hyperthermostable β-galactosidase enzyme in chitosan beads for efficient galacto-oligosaccharide production. J Dairy Sci. (2018) 101:1–10. 10.3168/jds.2017-13770 29397172

[83] Lopez-Moya F Kowbel D Nueda MJ Palma-Guerrero J Glass NL Lopez-Llorca LV. Neurospora crassa transcriptomics reveals oxidative stress and plasma membrane homeostasis biology genes as key targets in response to chitosan. Mol BioSyst. (2016) 12:391–403. 10.1039/C5MB00649J 26694141 PMC4729629

[84] Martínez B Rodríguez A Kulakauskas S Chapot-Chartier MP. Cell wall homeostasis in lactic acid bacteria: threats and defences. FEMS Microbiol Rev. (2020) 44:538–64. 10.1093/femsre/fuaa021 32495833 PMC7476776

[85] Kulikov SN Tikhonov VE Bezrodnykh EA Lopatin SA Varlamov VP. Comparative evaluation of antimicrobial activity of oligochitosans against *Klebsiella pneumoniae*. Bioorg Khim. (2015) 41:67–73. 10.1134/S1068162015010100 26050473

[86] Pham ML Tran AM Mathiesen G Nguyen HM Nguyen TH. Cell wall anchoring of a bacterial chitosanase in *Lactobacillus plantarum* using a food-grade expression system and two versions of an LP × TG anchor. Int J Mol Sci. (2020) 21:3773. 10.3390/ijms21113773 32471049 PMC7312796

[87] Kreling V Falcone FH Herrmann F Kemper L Amiteye D Cord-Landwehr S. High molecular/low acetylated chitosans reduce adhesion of *Campylobacter jejuni* to host cells by blocking JlpA. Appl Microbiol Biotechnol. (2024) 108:13. 10.1007/s00253-024-13000-0 38265503 PMC10810038

[88] Kurchenko V Halavach T Shramko M Lodygin A Evdokimov I. Application of chitosan for fermented dairy products with *Lactobacillus delbrueckii* subsp. Bulgaricus manufacturing. In:Kurchenko V Lodygin A Machado da Costa RM Samoylenko I, editors. ICAETT (2021) Lecture Notes in Networks and Systems, Vol. 408. Cham: Springer Nature Switzerland AG (2022). p. 167–75. 10.1007/978-3-030-96641-6_20

[89] Albadran HA Chatzifragkou A Khutoryanskiy VV Charalampopoulos D. Stability of probiotic *Lactobacillus plantarum* in dry microcapsules under accelerated storage conditions. Food Res Int. (2015) 74:208–16. 10.1016/j.foodres.2015.05.016 28411985

[90] Mesquita ARCd Silveira LPdM Cruz Filho IJD Lima VFd Silveira Filho VDM Araujo AA. Metabolism and physiology of Lactobacilli: a review. J Env Anal Progr. (2017) 2:115–36. 10.24221/jeap.2.2.2017.1202.115-124

[91] Zielińska K Fabiszewska A Swiatek M Szymanowska-Powałowska D. Evaluation of the ability to metabolize 1,2-propanediol by heterofermentative bacteria of the genus Lactobacillus. Electr J Biotechnol. (2017) 26:60–3. 10.1016/j.ejbt.2017.01.00233309357

[92] Zhang C Brandt MJ Schwab C Gänzle MG. Propionic acid production by cofermentation of *Lactobacillus buchneri* and Lactobacillus diolivorans in sourdough. Food Microbiol. (2010) 27:390–5. 10.1016/j.fm.2009.11.019 20227604

[93] Johanningsmeier SD Feeters RF. Metabolism of lactic acid in fermented cucumbers by *Lactobacillus buchneri* and related species, potential spoilage organisms in reduced salt fermentationsq. Food Microbiol. (2013) 35:129–35. 10.1016/j.fm.2013.03.004 23664264

[94] Siddiqui SA Bahmid NA Taha A Khalifa I Khan S Rostamabadi H. Recent advances in food applications of phenolic-loaded micro/nanodelivery systems. Crit Rev Food Sci Nutr. (2022) 63:8939–59. 10.1080/10408398.2022.2056870 35426751

[95] Atwaa ESH Shahein MR Raya-Álvarez E Abd El-Sattar ES Hassan MAA Hashim MA. Assessment of the physicochemical and sensory characteristics of fermented camel milk fortified with *Cordia myxa* and its biological effects against oxidative stress and hyperlipidemia in rats. Front Nutr. (2023) 10:1130224. 10.3389/fnut.2023.1130224 37229477 PMC10203225

[96] Partanen L Marttinen N Alatossava T. Fats and fatty acids as growth factors for *Lactobacillus delbrueckii*. System Appl Microbiol. (2001) 24:500–6. 10.1078/0723-2020-00078 11876356

[97] Yerlikaya O Gucer L Akan E Meric S Aydin E Kinik O. Benzoic acid formation and its relationship with microbial properties in traditional Turkish cheese varieties. Food Biosci. (2021) 41:101040. 10.1016/j.fbio.2021.101040

[98] Bartáková K Vorlová L Dluhošová S Borkovcová I Bursová Š Pospíšil J. Effect on benzoic acid production of yoghurt culture and the temperatures of storage and milk heat treatment in yoghurts from cow, goat and sheep milk. Foods. (2021) 10:1535. 10.3390/foods10071535 34359405 PMC8303823

[99] García-Díez J Saraiva C. Use of starter cultures in foods from animal origin to improve their safety. Int J Environ Res Public Health. (2021) 18:2544. 10.3390/ijerph18052544 33806611 PMC7967642

[100] Deepthi BV Poornachandra RK Chennapa G Naik MK Chandrashekara KT Sreenivasa MY. Antifungal attributes of *Lactobacillus plantarum* MYS6 against fumonisin producing *Fusarium proliferatum* associated with poultry feeds. PLoS ONE. (2016) 11:e0155122. 10.1371/journal.pone.0155122 27285317 PMC4902316

[101] Siedler S Balti R Neves AR. Bioprotective mechanisms of lactic acid bacteria against fungal spoilage of food. Curr Opin Biotechnol. (2019) 56:138–46. 10.1016/j.copbio.2018.11.015 30504082

[102] Szajnar K Znamirowska A Kuzniar P. Sensory and textural properties of fermented milk with viability of *Lactobacillus rhamnosus* and *Bifidobacterium animalis* ssp. Lactis Bb-12 and increased calcium concentration. Int J Food Propert. (2020) 23:582–98. 10.1080/10942912.2020.1748050

[103] Zobkova ZS Lazareva EG Semipyatniy VK. Methodological approach to designing fermented dairy products with optimal biological value. Foods. (2022) 11:114. 10.3390/foods11010114 35010240 PMC8750144

[104] Vlasenko I Bandura V Semko T Fialkovska L Ivanishcheva O Palamarchuk V. Innovative approaches to the development of a new sour milk product. Potr S J F Sci. (2021) 15:970–81. 10.5219/1688

[105] Han N Park SY Kim SY Yoo MY Paik HD Lim SD. Short communication: change of naturally occurring benzoic acid during skim milk fermentation by commercial cheese starters. J Dairy Sci. (2016) 99:8633–7 10.3168/jds.2016-10890 27592433

