# Supplementary figures and images for: Chitosan and its derivatives regulate lactic acid synthesis during milk fermentation
Source: Front Nutr. 2024 Sep 16;11:1441355. doi: 10.3389/fnut.2024.1441355 (PMC11439701; doi:10.3389/fnut.2024.1441355)

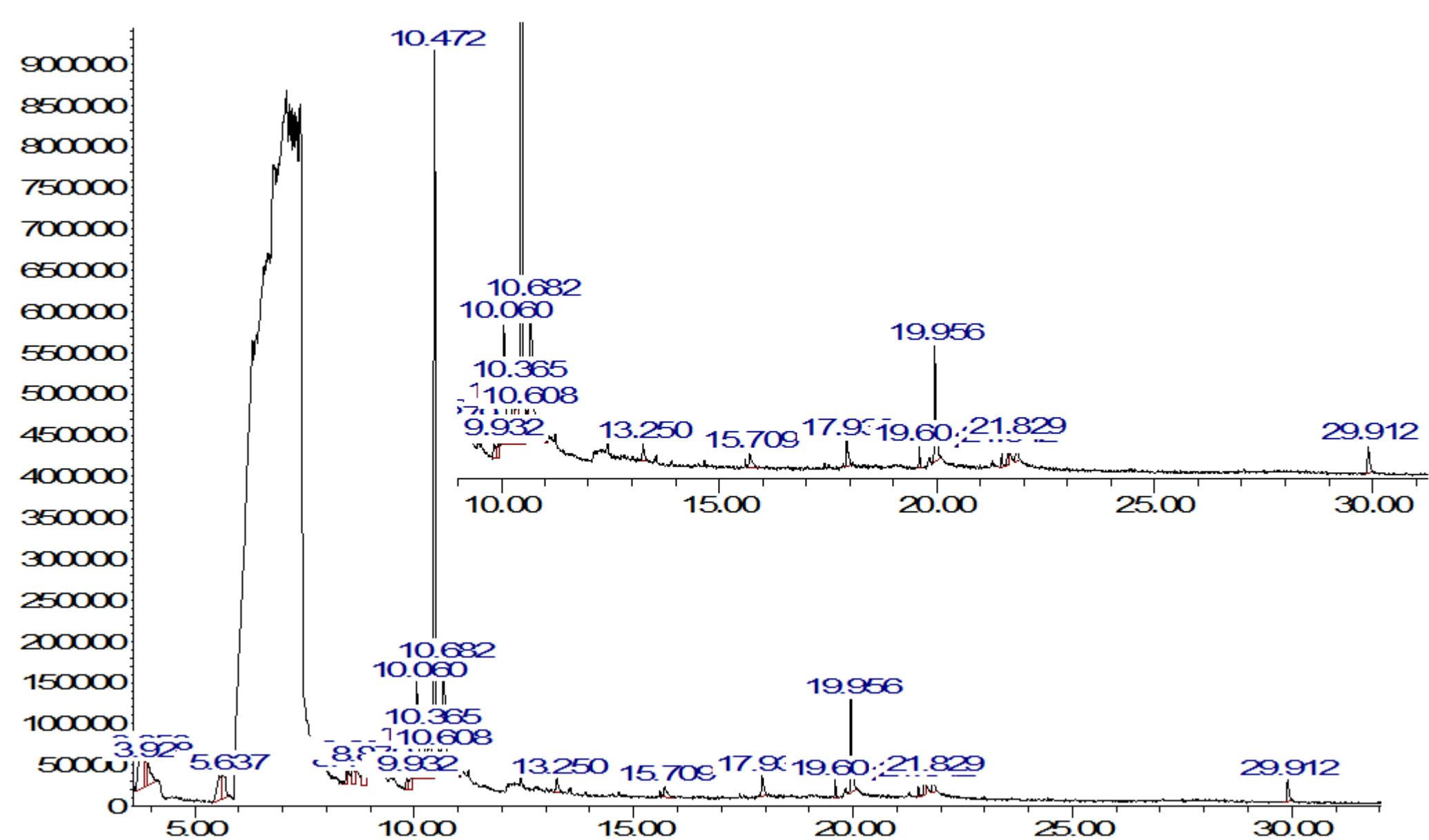

Supplement: Supplementary Figure 1 — A typical GC-MS chromatogram of an extract from fermented dairy product on the 17th day of storage. [file Image_1.pdf]

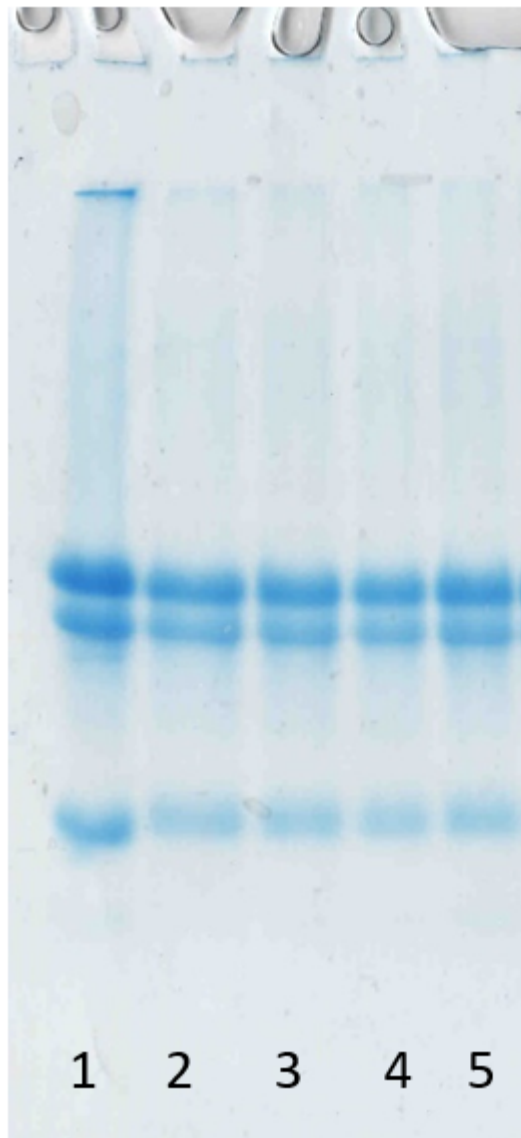

caseins

$\beta$ -Ig

$\alpha$ -Ig

1

2

3

4

5

Supplement: Supplementary Figure 2 — SDS-electrophoregram of proteins of fermented dairy product on the 17th day of storage: control sample (5), 0.01% CH (4), 0.0075% CH (3), 0.0025% CH (2), original milk (1). [file Image_2.pdf]
